# Crystal structure of mouse DXO in complex with the UDP-*N*-acetylglucosamine cap and molecular mechanism for the decapping reactions

**DOI:** 10.1093/nar/gkag521

**Published:** 2026-05-27

**Authors:** Najeeb Ullah, Selom K Doamekpor, Liang Tong

**Affiliations:** Department of Biological Sciences, Columbia University, New York, NY 10027, United States; Department of Biological Sciences, Columbia University, New York, NY 10027, United States; Department of Biological Sciences, Columbia University, New York, NY 10027, United States

## Abstract

Noncanonical metabolite 5′ caps have recently been identified on RNAs, and the DXO/Rai1 family of enzymes can remove these caps in eukaryotes. While the binding modes of NAD, FAD and dephospho-CoA (dpCoA) caps in the active site of mouse DXO have been determined, how DXO recognizes the UDP-glucose (UDP-Glc) and UDP-*N*-acetylglucosamine (UDP-GlcNAc) caps is not known. In addition, the molecular mechanism by which DXO catalyzes the decapping reactions is still poorly understood, especially the location of the water/hydroxide that attacks the scissile phosphate to initiate the decapping. Here we report the crystal structure of mouse DXO in complex with UDP-GlcNAc at 1.8 Å resolution. The binding mode of the compound explains why DXO removes the entire cap from RNA. We have also determined the structures of mouse DXO in complex with purine oligonucleotides, pA5 and pGGGUU. Most importantly, we have produced a model of DXO in a catalytically competent complex with substrates, revealing that a water/hydroxide coordinated to the first metal ion is the nucleophile that attacks the scissile phosphate. The conformation of the scissile phosphate is similar to an alternate conformer of the 5′ phosphate in pA5, which provides experimental support for the modeled substrate complex.

## Graphical Abstract

**Graphical Abstract:**
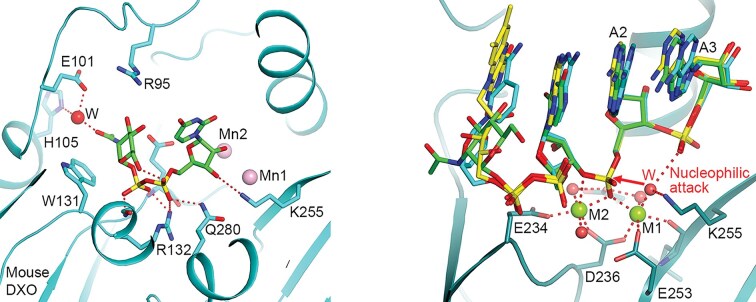

## Introduction

The co-transcriptional acquisition of a 5′ cap is an obligate event in the maturation of messenger RNA precursors (pre-mRNAs) in eukaryotes. The cap is generated in three steps: conversion of the 5′ triphosphate group of the primary transcript from RNA polymerase II (Pol II) to a diphosphate, addition of a GMP molecule through a 5′-5′ linkage, and finally the methylation of the N^7^ position of the guanine [1]. This N^7^-methylguanosine (m^7^G) cap is critical for the splicing, 3′-end processing, stability, export, and translation of mRNAs [2].

The DXO/Rai1 family of enzymes consists of homologs of mammalian DXO and fungal Rai1 and Dxo1. They can have RNA 5′ pyrophosphohydrolase (PPH) activity, being able to convert the 5′ triphosphate group to a monophosphate [3, 4]. In addition, these enzymes can remove the unmethylated cap, producing GpppN and 5′ monophosphate RNA [5]. Therefore, the DXO/Rai1 enzymes can remove intermediates from the capping process, enabling the RNA body to be degraded by 5′-3′ exoribonucleases (which require a 5′ monophosphate). This establishes for the first time a quality surveillance mechanism for RNA 5′ capping, with incompletely capped RNAs being degraded by DXO/Rai1 and 5′-3′ exoribonucleases. In addition, DXO, Dxo1, and selected other members of the DXO/Rai1 family also possess 5′-3′ exoribonuclease activity, hence the decapping exonuclease (DXO) name [6, 7].

Besides the canonical m^7^G cap, recent studies have revealed that mRNAs can carry noncanonical, metabolite caps, such as NAD, FAD, dephospho-CoA (dpCoA), UDP-glucose (UDP-Glc) and UDP-*N*-acetylglucosamine (UDP-GlcNAc) [8–18]. The noncanonical caps can affect the stability, metabolism, and translation of the mRNAs, although their exact cellular functions are still not fully understood. The DXO/Rai1 enzymes can also remove these caps, through their deNADing [8], deFADding and deCoAping activities [19]. The binding modes of NAD, FAD and dpCoA in the active site of mouse DXO have been determined [8, 19], revealing how the enzyme can remove the entire metabolite cap from the RNA. The active site of DXO/Rai1 enzymes contains two metal ions (magnesium or manganese), and the scissile phosphate is bound by these metal ions. Conserved residues in six motifs (I through VI) [4] coordinate the metal ions and participate in substrate recognition and subsequent catalysis.

The noncanonical metabolite caps are also found on bacterial RNAs [20–22]. They can also be capped with Np(n)N alarmones [23–25], which have been found on eukaryotic RNAs as well [26]. The removal of these caps in bacteria is mediated by the Nudix family enzymes [27, 28], which also catalyze decapping in animals and plants [29–31]. 5′-3′ exoribonucleases can also have deNADding [32] or deFADding [33] activity. Hepatitis C virus RNA carries a 5′ FAD cap [34], underscoring the widespread relevance of noncanonical metabolite capping.

**Figure 1.**
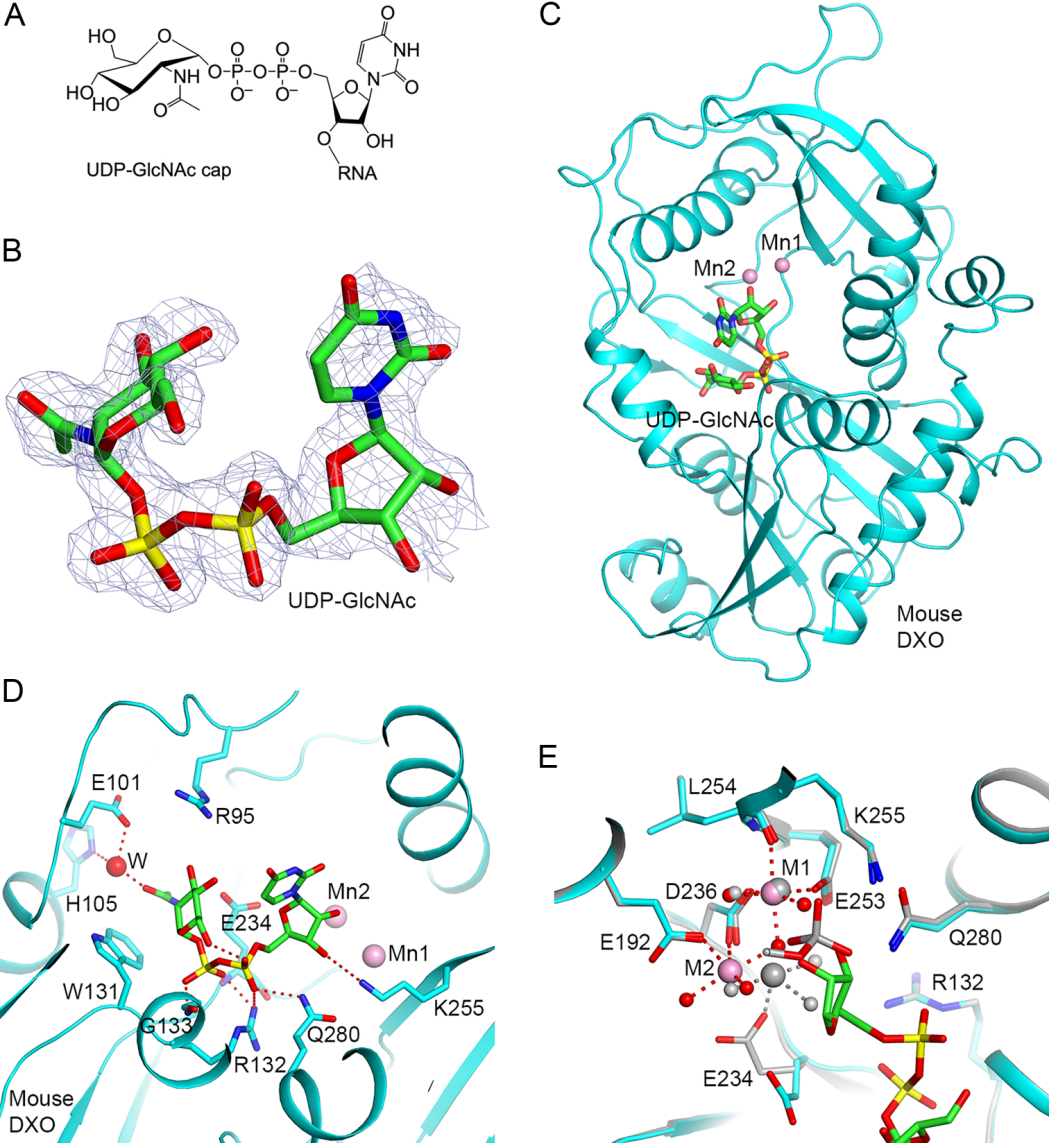
Crystal structure of mouse DXO in complex with UDP-GlcNAc. (**A**) Chemical structure of UDP-GlcNAc-capped RNA. (**B**) Simulated annealing omit 2F_o_–F_c_ electron density at 1.8 Å resolution for UDP-GlcNAc, contoured at 1σ. (**C**) Overall structure of wild-type mouse DXO (cyan) in complex with UDP-GlcNAc (in green for carbon atoms). The two Mn^2+^ ions are shown as spheres (pink). (**D**). Detailed interactions between UDP-GlcNAc and mouse DXO. Hydrogen-bonding interactions are drawn as dashed lines (red), and a water molecule is shown as a sphere (red). (**E**) A distinct binding mode for the two metal ions in the UDP–GlcNAc complex (in color). An overlay of the structure of the complex with the pU5 product (in gray). Residues involved in metal ion binding are shown as sticks. The metal ions are shown as large spheres and labeled, and water molecules are shown as small spheres (red). Coordination interactions between the metal ions and the ligands are indicated with the dashed lines.

RNAs with UDP-Glc and UDP-GlcNAc caps have been identified in bacteria [35] and in mammalian cells [36]. Human Nudix enzyme Nudt5 can remove UDP-GlcNAc caps [35]. Mammalian DXO and yeast Rai1 can also remove these noncanonical caps [36], although it is not known how they are recognized by these enzymes. We have determined the crystal structure of mouse DXO in complex with UDP-GlcNAc (Fig. 1A) at 1.8 Å resolution, revealing how the enzyme removes the entire cap from the RNA. In addition, we have determined the structures of mouse DXO in complex with pA5 and pGGGUU oligonucleotides, which are products of the decapping reaction and show how the bulkier purine bases are accommodated in the DXO active site. By combining experimental observations on the binding modes of the caps and products and AlphaFold 3 predictions, we have produced a model for DXO in a catalytically competent complex with substrates, providing atomic insights into the decapping reaction.

## Materials and methods

### Protein expression and purification

His-tagged full-length wild-type mouse DXO in pET28a were used to transform *E. coli* BL21 (DE3) Rosetta cells. The protein was expressed and purified as previously reported [3]. Briefly, DXO expression was induced with 0.3 mM IPTG at 18°C overnight and purified with Ni-NTA Superflow (Qiagen) and gel filtration (Sephacryl S-300) chromatography (in running buffer containing 20 mM Tris (pH 7.5), 250 mM NaCl, and 2 mM DTT). The protein was supplemented with 5% (v/v) glycerol and concentrated to 10 mg/ml before being frozen in liquid nitrogen.

### Crystallization, data collection and structure determination

Mouse DXO crystals were obtained using the hanging-drop vapor diffusion method at 20°C with a reservoir solution containing 21–26% (w/v) PEG 3350. For the UDP–GlcNAc complex, crystals were soaked in 20 mM MnCl_2_ for 10 mins followed by soaking in 10 mM UDP-GlcNAc (sodium salt) solution in a new drop for additional 30 mins at 20°C in buffer containing 25% (w/v) PEG 3350 and 0.1 M Tris (pH 6.8). DXO can use both Mg^2+^ and Mn^2+^ for catalysis, and we tried both metal ions in our experiments and selected the better quality dataset for presentation here.

For the pA5 complex, crystals were soaked in 20 mM MgCl_2_ for 10 mins, then the soaked crystals were transferred to a new drop containing 5 mM pA5 for 30 mins in a buffer containing 25% (w/v) PEG 3350 and 0.1 M Tris (pH 6.8).

For the pGGGUU complex, crystals were soaked in 10 mM pGGGUU and 20 mM MgCl_2_ overnight. Crystals were cryo-protected with 25% (w/v) PEG 3350 and 25% (v/v) ethylene glycol before being flash frozen in liquid nitrogen for diffraction analysis and data collection at 100 K.

X-ray diffraction data were collected at the Advanced Photon Source (APS) beamline 24-ID-E, and the diffraction images were processed and scaled using the XDS program [37]. The crystals are isomorphous to those reported earlier [3]. The structure refinement was initiated with a rigid-body refinement in Phenix [38] with DXO (PDB entry 4J7L) as the initial model. Further structure refinement was performed using PHENIX, and the atomic model was built with the Coot program [39]. No electron density was observed for residues at the N (1–26, together with the His tag) and C (385–397) termini of mouse DXO, and they were not included in the atomic model. The crystallographic information is summarized in Table 1.

## Results

### Structural basis for the recognition of UDP-GlcNAc by mouse DXO

To elucidate the molecular mechanism for how UDP-GlcNAc is recognized by DXO, we soaked crystals of wild-type mouse DXO with the compound (at 10 mM concentration) and catalytic divalent cations (magnesium or manganese, 20 mM) before freezing the crystals in liquid nitrogen. Several X-ray diffraction datasets were collected on the soaked crystals. Electron density for the diphosphate of UDP was observed in most of the datasets, but the density for uridine-ribose and GlcNAc showed variable quality among the datasets. The structure presented here had the best electron density for GlcNAc and uridine (Fig. 1B), but the density was still weaker than that observed for DXO residues in the active site region. This suggests that the two groups have some flexibility in the complex with mouse DXO. The refined atomic model has excellent agreement with the X-ray diffraction data and the expected bond lengths, bond angles and other geometric parameters (Table 1).

**Table 1.** Summary of crystallographic information

Structure	Mouse DXO +UDP-GlcNAc	Mouse DXO + pA5	Mouse DXO + pGGGUU
Data Collection			
Space group	*P*2_1_	*P*2_1_	*P*2_1_
Cell dimensions			
*a, b, c* (Å)	49.9, 88.0, 53.6	50.0, 88.2, 53.1	46.4, 88.0, 50.2
α, β, γ (°)	90, 113.0, 90	90, 113.2, 90	90, 114.4, 90
Resolution (Å)^1^	49.3–1.8(1.85–1.8)	48.8–1.59(1.63–1.59)	42.2–1.6(1.70–1.6)
*R*_merge_ (%)	14.9 (88.2)	6.9 (64.3)	3.2 (41.0)
I/σI	11.8 (2.7)	13.5 (2.8)	14.3 (2.0)
CC_1/2_	0.995 (0.722)	0.998(0.80)	0.999 (0.733)
Completeness (%)	98.2 (96.6)	98.8 (98.6)	94.8 (93.8)
No. of reflections	38 997	56 497	90 196
Redundancy	3.9 (3.6)	4.1 (4.0)	2.0 (1.9)
Refinement			
Resolution (Å)	49.3–1.8(1.85–1.8)	48.8–1.59(1.63–1.59)	42.2–1.6(1.64–1.6)
*R*_work_ (%)	16.3 (27.2)	18.1 (28.3)	18.4 (30.0)
*R*_free_ (%)	20.6 (31.8)	18.9 (27.0)	21.1 (33.7)
Number of atoms	3371	3282	3332
Protein	2901	2901	2909
Ligand	39	94	98
Ion	2	2	2
Water	428	289	323
B-factors (Å^2^)	26.4	24.3	26.2
Protein	24.6	23.1	25.0
Ligand	35.9	33.9	34.4
Ion	23.3	13.5	13.7
Water	35.7	32.8	34.6
r.m.s.d.			
Bond lengths (Å)	0.006	0.002	0.006
Bond angles (°)	0.87	0.50	0.86

1. The numbers in parentheses are for the highest resolution shell

UDP-GlcNAc is situated in the active site of DXO (Fig. 1C). The uridine base does not have any direct interactions with DXO (Fig. 1D), consistent with its flexibility in the complex. The ribose of UDP is positioned near the two metal ions, with the hydroxyls ∼3.6 Å away from them, and therefore, the ribose hydroxyls are not direct ligands to the metal ions. The 3′ hydroxyl is within hydrogen-bonding distances of the side chains of Lys255 (motif IV) and Gln280 (motif V). Both phosphates of UDP have interactions with the side chains of Arg132 (motif I). In addition, the α phosphate is hydrogen-bonded to the side chain of Gln280 (motif V), and the β phosphate is hydrogen-bonded to the Gly133 amide nitrogen at the N-terminus of helix αB and the E234 amide nitrogen (motif III, a ligand to the second metal ion).

The glucosamine ring of the bound UDP-GlcNAc is in a chair conformation and is positioned perpendicular to the side chain of Trp131 (Fig. 1D). The C6 hydroxyl is hydrogen-bonded to the α phosphate of UDP, and the C3 hydroxyl is within 3.5 Å of the guanidinium group of Arg95. The C4 hydroxyl has no direct contacts with DXO. The C2 acetyl group is placed next to the Trp131 side chain, with the planes of the two groups being in parallel, thereby showing π-stacking interactions. The carbonyl oxygen is hydrogen-bonded to the side chains of Glu101 and His105 through a water molecule.

The UDP-GlcNAc molecule lacks a phosphate on the 3′ hydroxyl of the UDP, which would mimic the connection to the body of the RNA. Nonetheless, the binding mode of this compound indicates that the 3′ phosphate would be placed next to the metal ions. Therefore, the structure demonstrates that this 3′ phosphate would be the scissile phosphate of UDP-GlcNAc capped RNAs, consistent with the biochemical data [36].

This structure contains two Mn^2+^ ions in the active site. The first metal ion has the same binding mode as that observed earlier [3, 7], interacting with the main-chain carbonyl oxygen of Leu254 and the side chains of Asp236 (motif III) and Glu253 (motif IV) (Fig. 1E). The 5′ phosphate of the pU5 product provides two ligands to this metal ion, which are replaced by waters in the UDP–GlcNAc complex, as it lacks a phosphate group here.

In contrast, the second metal ion is located at a different position in the UDP–GlcNAc complex, 2 Å away from the position of the second metal ion observed earlier with the pU5 oligo [7]. In fact, this metal ion is located close to the position of one of the water ligands to the second metal ion in the earlier structure. It is directly coordinated to Glu192 (motif II), rather than Glu234 (motif III), while the reverse is true in the earlier structure. This metal ion in the current structure has partial occupancy (∼0.6) based on the crystallographic analysis, and its coordination sphere is not complete either. The observation of this new metal binding position is probably linked to the fact that the UDP-GlcNAc ligand here does not have a 3′ phosphate group, and it remains to be seen whether this new metal position could also be involved in the catalysis by DXO. The binding of the two metal ions should be mutually exclusive because of their close separation, and the observation of these two binding modes for the second metal ion does not suggest that DXO catalysis involves three metal ions.

### Comparison to the binding modes of other metabolite caps

**Figure 2.**
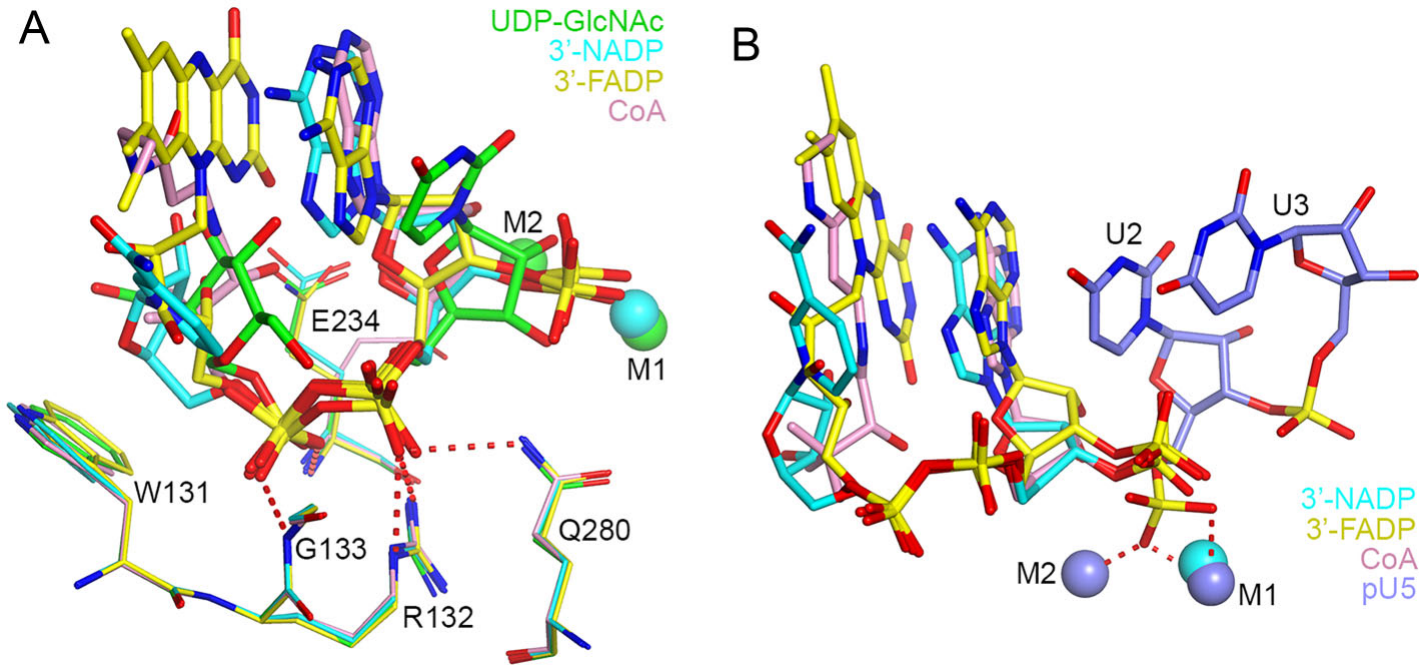
Comparison of the binding modes of noncanonical metabolite caps in DXO. (**A**) Overlay of the binding modes of UDP-GlcNAc (green for carbon atoms), 3′-NADP (cyan), 3′-FADP (yellow) and CoA (pink). The metal ions are shown as spheres, in the same color as the metabolite cap. Residues in DXO are shown as thin sticks. (**B**) Overlay of the binding modes of 3′-NADP (cyan), 3′-FADP (yellow), CoA (pink) and pU5 (slate). The 3′ phosphate groups of NADP, FADP and CoA are in a different position compared to the 5′ phosphate of pU5.

With the structure of the UDP–GlcNAc complex reported here, the binding modes of four noncanonical metabolite caps in mouse DXO are now known: NAD [8], FAD [19], dpCoA [19], and UDP-GlcNAc. A comparison of these binding modes provides further molecular insights into the recognition of these noncanonical caps by DXO. A conserved feature among all four caps is the pyrophosphate group, and it has essentially the same binding mode in the four structures (Fig. 2A). Arg132 (motif I), Gln280 (motif V) and two main-chain amide groups have crucial interactions with the phosphates, defining a conserved recognition site. This pyrophosphate belongs to a nucleotide (ADP or UDP) that is positioned just 5′ to the cleavage site. It may be expected that this conserved binding site also recognizes the 5′ phosphate group of the RNA substrate for the 5′-3′ exonuclease activity, and the 5′ triphosphate group for the pyrophosphohydrolase (PPH) activity of DXO.

The moiety just prior to the pyrophosphate in these metabolite caps has extensive interactions with the side chain of Trp131, which is conserved as Trp/Tyr/Phe in most DXO/Rai1 homologs, suggesting that this is another important region of the DXO active site. Additional components of these metabolite caps establish other interactions with DXO, and some also have π-stacking and/or van der Waals interactions (FAD and dpCoA) with the base of the nucleotide (Fig. 2A). The nicotinamide ring is not π-stacked with the adenine base in the observed binding mode for NAD.

The studies with the NAD, FAD and dpCoA caps introduced a phosphate group at the 3′ position of the ribose, giving rise to 3′-NADP (NADP in short here), 3′-FADP (FADP) and CoA. This 3′ phosphate would be the 5′ phosphate group of the RNA body and also the scissile phosphate group of the substrate. On the other hand, the structures of the DXO complexes with these three caps contain either one Ca^2+^ ion (a noncatalytic metal ion, NADP complex) or no metal ions (due to the use of an active site E192S/E234Q/E253Q mutant). While the phosphate group is at nearly the same position in the three structures, this position is different compared to that observed in the pU5 complex, and the phosphate would not have optimal interactions with the metal ions even if they were bound (Fig. 2B).

The binding mode of the uridine base and ribose in UDP-GlcNAc is very different, with the ribose moving toward the location of the 3′ phosphate group (Fig. 2A). The binding mode of this segment of UDP-GlcNAc is likely affected by the absence of this phosphate group.

### Binding modes of the pA5 and pGGGUU products

**Figure 3.**
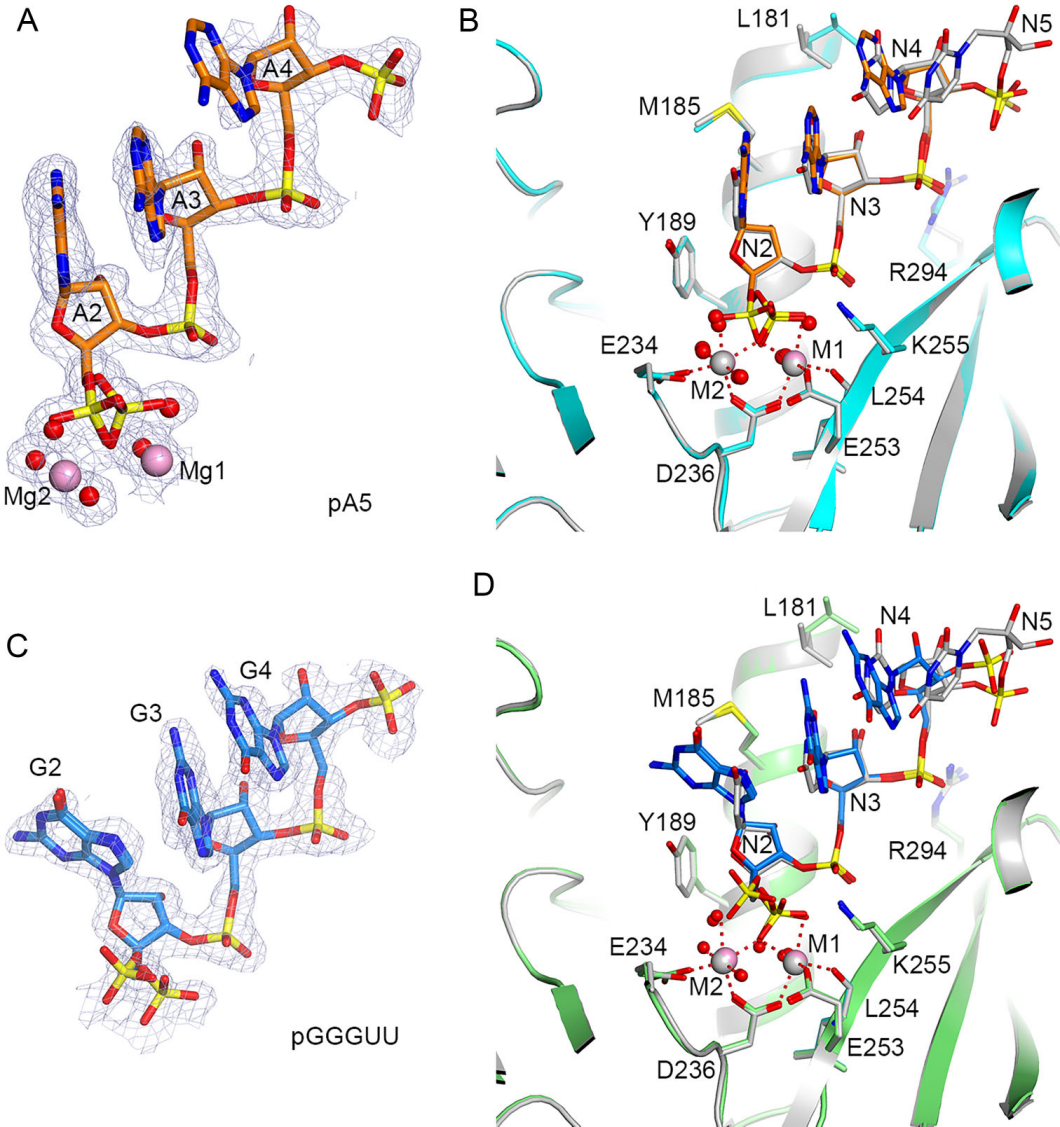
Crystal structures of mouse DXO in complex with pA5 and pGGGUU. (**A**) Simulated annealing omit 2F_o_–F_c_ electron density at 1.59 Å resolution for pA5, contoured at 0.8σ. (**B**) Overlay of the active site region of mouse DXO in complex with pA5 (in color) with that in complex with pU5 (gray). (**C**) Simulated annealing omit 2F_o_–F_c_ electron density at 1.6 Å resolution for pGGGUU, contoured at 0.8σ. (**D**). Overlay of the active site region of mouse DXO in complex with pGGGUU (in color) with that in complex with pU5 (gray).

We have shown earlier how the pU5 product RNA is recognized by DXO [7]. To understand how the bulkier purine bases are accommodated in the DXO active site, we have determined the structures of mouse DXO bound to pA5 and pGGGUU oligonucleotides. Our earlier studies showed that the active site of DXO could accommodate at most 5 nucleotides in the product RNA, and the 5′ phosphate of the RNA would also be the scissile phosphate of the substrate [7]. For the pA5 complex, crystals of wild-type mouse DXO were soaked with a pA5 oligonucleotide (5 mM) and Mg^2+^ or Mn^2+^ ions (20 mM) before being flash frozen. Several diffraction datasets were collected, and the one showing the best electron density for the oligo was selected. This led to a structure of mouse DXO in complex with pA5 and two Mg^2+^ ions at 1.59 Å resolution (Table 1). Only the first three nucleotides and the 5′ phosphate of the fourth nucleotide were observed in the electron density (Fig. 3A), and the other nucleotides were disordered and/or removed by the exonuclease activity of DXO during the soaking.

The overall binding mode of pA5 was very similar to that of the first three nucleotides of pU5, and the positions of the two metal ions are similar as well (Fig. 3B). The bulkier adenine bases could be accommodated without introducing large changes in their binding mode or in DXO. As with the pU5 structure, the pA5 nucleotides are numbered from A2 to A5, with residue 1 being the cap/nucleotide in the substrate that is removed by DXO.

The 5′ phosphate of pA5 shows two-fold disorder (Fig. 3B). One of these conformers (40% occupancy) is essentially the same as that observed earlier in the pU5 complex. In this conformer, one of the terminal oxygen atoms of the phosphate is a bridging ligand to the two metal ions, while another is a ligand to the first metal ion. For the other conformer (60% occupancy), the phosphate group has rearranged such that one of its oxygen atoms is a ligand to the second metal ion instead, although an oxygen atom is still the bridging ligand between the two metal ions. We will refer to this as the alternate conformer here. A water molecule occupies the ligand position when the phosphate is in the other conformer.

For the pGGGUU complex, we soaked crystals of wild-type mouse DXO with a pGGGUU oligo and Mg^2+^ overnight, and the resulting crystallographic analysis revealed only pG3 and the phosphate of the following nucleotide. We also observed two-fold disorder for the 5′ phosphate of this oligo (Fig. 3C). One binding mode was similar to that observed with pU5, while the other had the phosphate in a distinct position, which we will refer to as the away conformer. In this way, the phosphate no longer provides an oxygen atom as the bridging ligand between the two metal ions, and it instead can only provide a ligand to the second metal ion (Fig. 3D). Therefore, this way conformer is different from the alternate conformer observed with pA5 (Fig. 3B).

The guanine base of the first nucleotide of pGGGUU rotates by ∼80 ° relative to that of pU5 or pA5 to avoid a steric clash with the side chain of Met185, so that this base is nearly perpendicular to the base of the second nucleotide. This conformational change is likely driven by the presence of the 2-amino group on guanine. The binding modes of the second and third nucleotides of pGGGUU are similar to those of pU5. The loss of base stacking for the first guanine base could be consistent with the weaker activity of DXO toward this substrate that we observed earlier [40].

### A model of DXO in a catalytically competent complex with substrate

To fully understand how DXO removes the 5′ cap, especially the location of the water/hydroxide that is expected to attack the scissile phosphate to initiate the hydrolysis, a structure of its complex with a substrate or a close substrate analog in a catalytically competent binding mode is needed. We used a non-hydrolyzable RNA oligo earlier, which provided insight into how a substrate could bind in the active site [7]. However, the sulfur atom in the phosphorothioate linkage (to prevent hydrolysis) perturbed the binding mode of the scissile group of the substrate. In addition, there was only one metal ion (a noncatalytic Ca^2+^) in the structure. Therefore, information was still lacking as to how the hydrolysis was initiated. Our many subsequent attempts at determining a structure of DXO in complex with a substrate in a productive binding mode were not successful either.

**Figure 4.**
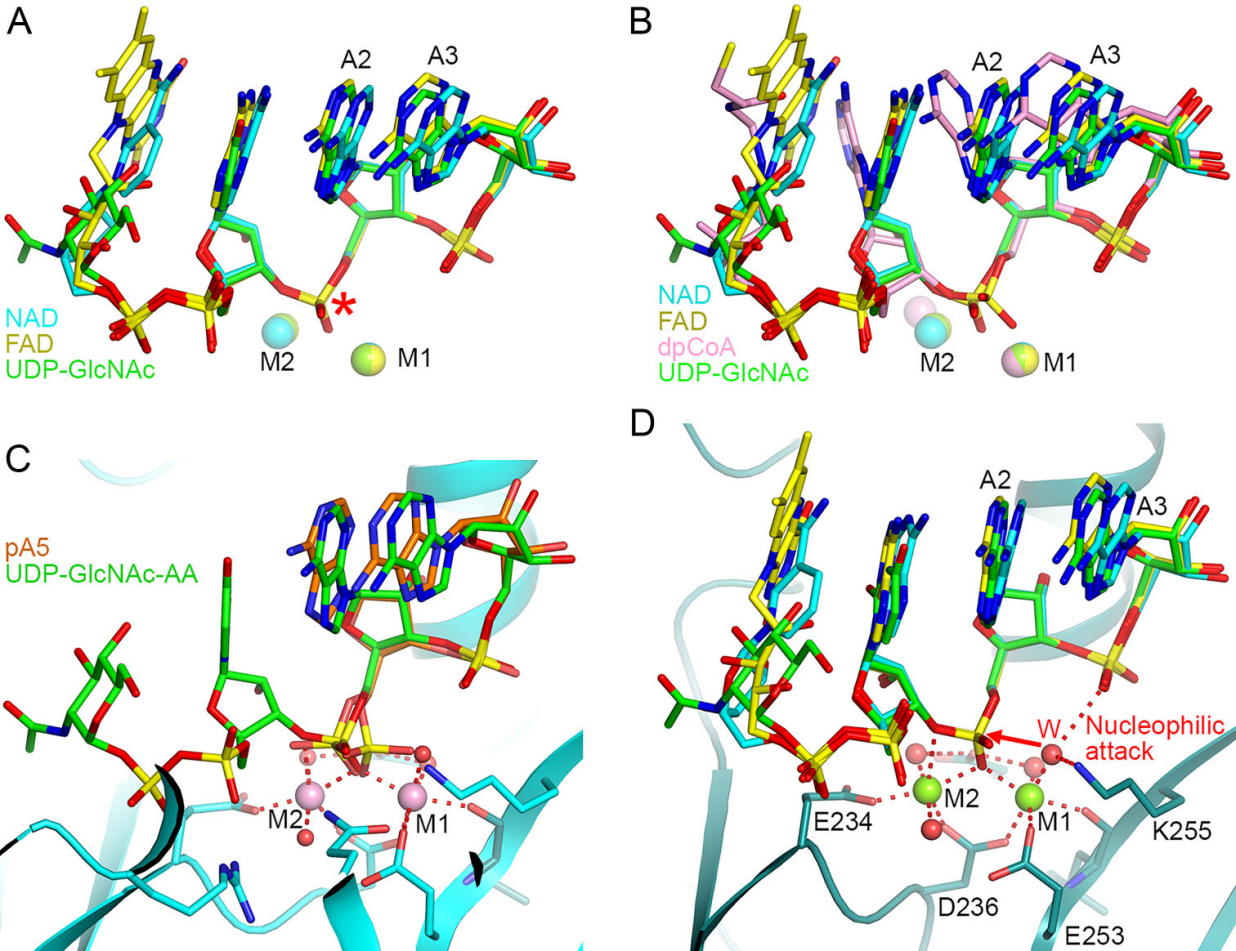
Model of DXO in a catalytically competent complex with substrate. (**A**) Overlay of the binding modes of NAD-AA (cyan), FAD-AA (yellow), UDP-GlcNAc-AA (green), and two Mg2 + ions (same color as the corresponding cap) in the mouse DXO active site as predicted by AlphaFold 3. The scissile phosphate is indicated with the red asterisk. (**B**) Overlay of the binding modes of NAD-AA (cyan), FAD-AA (yellow), dpCoA-AA (pink), UDP-GlcNAc-AA (green), and two Mg2 + ions in the mouse DXO active site as predicted by AlphaFold 3. (**C**) Overlay of the predicted binding mode of UDP-GlcNAc-AA (green) with the structure of mouse DXO (cyan) in complex with pA5 (orange). (**D**). Model of DXO in a catalytically competent complex with NAD-AA, FAD-AA and UDP-GlcNAc-AA substrate. The water ligand to the first metal ion (labeled W) is the nucleophile attacking the scissile phosphate (red arrow). The leaving group, 3′ hydroxide of the cap, is stabilized through interaction with the second metal ion.

With the availability of AlphaFold 3 [41], we used it to predict the binding modes of substrates in DXO. The substrates that we examined were 5′-cap-AA, with two adenine nucleotides as the body of the RNA. A model was generated for each of the four noncanonical metabolite caps, NAD, FAD, dpCoA and UDP-GlcNAc (with ipTM of 0.73–0.78). Two Mg^2+^ ions were also included for each prediction based on our observations on the binding of product RNAs [7]. The models for the NAD-, FAD- and UDP-GlcNAc-capped RNAs shared remarkable similarity in the binding modes of the substrates, especially the binding mode of the scissile phosphate and the positions of the two metal ions (Fig. 4A). The model for the dpCoA-capped RNA showed recognizable differences for the positions of the scissile phosphate, the second metal ion and the bases (Fig. 4B). It was not clear why the dpCoA substrate exhibited such differences, and this model will not be discussed further.

The position of the scissile phosphate in the models remarkably corresponded to that of the alternate conformer observed in the pA5 complex (Fig. 4C), providing experimental support for the validity of the models. Such a model is unlikely to be biased by prior structural information in the PDB, as there are no structures available for such complexes of DXO/Rai1 enzymes in the database at the moment.

Combining the AlphaFold3 models for the substrates NAD-AA, FAD-AA and UDP-GlcNAc-AA and the structure of the pA5 complex, we have built a model for the catalytic state of DXO (Fig. 4D). In this model, with the scissile phosphate interacting with the second metal ion, water would be bound to the first metal ion. This water is located at the correct position to initiate the nucleophilic attack on the scissile phosphate, directly opposite the leaving group and 2.8 Å from the phosphorus atom. Based on the model, the water is likely activated by the metal ion and possibly the side chain of Lys255. One of the terminal oxygen atoms of nucleotide A3 in the substrate is also located near this water, and it may make some contribution to the activation as well. The 3′ hydroxide leaving group is stabilized by its interaction with the second metal ion.

## Discussion

Our studies have revealed the binding modes of four noncanonical metabolite caps as well as purine and pyrimidine oligonucleotides in the DXO active site. Common features observed for these binding modes explain why DXO/Rai1 enzymes remove the entire 5′ cap from the RNA, which is driven by the presence of a conserved (pyro)phosphate recognition site 5′ to the cleavage site. In fact, with an RNA substrate that has a 5′ hydroxyl group, DXO recognizes the phosphate between the first and second nucleotide and releases a 5′ hydroxyl dinucleotide from the substrate [40].

Our model for the DXO–substrate complex reveals for the first time the location of the water/hydroxide that initiates the decapping reaction and the binding mode of the substrate just prior to the reaction. In the decapping reaction by Nudix enzymes, a water/hydroxide that bridges two metal ions in the active site is the nucleophile that attacks the scissile phosphate [29, 42]. In comparison, the nucleophile in DXO appears to be a water/hydroxide coordinated to only one metal ion, and there is no bridging water/hydroxide in the active site (Fig. 4D). In many other two-metal-ion catalysis reactions, it is also a water/hydroxide coordinated to only one metal ion that is the nucleophile [43].

Remarkably, the productive binding mode of the scissile phosphate is also observed as an alternate conformer for the 5′ phosphate of the pA5 product, which would be the scissile phosphate in the substrate. This offers experimental support for the modeled binding mode of the substrate. The two conformers of the 5′ phosphate of pA5 represent the product and substrate states of the scissile phosphate. We also tried to predict the binding mode of an RNA substrate with the canonical cap, (m^7^)GpppG-AA, but the resulting models had low confidence. It is likely that DXO catalyzes the canonical decapping reaction using a similar mechanism, although further experiments will be needed to confirm this.

## Data Availability

The atomic coordinates and X-ray diffraction data have been deposited at the Protein Data Bank, with accession codes 11AP (UDP-GlcNAc complex), 11AO (pA5 complex), and 10YZ (pGGGUU complex).

## Notes

Present address: Department of Biochemistry and Molecular Biology, Thomas Jefferson University, Philadelphia, PA 19107, United States
